# Abdominal Obesity–Metabolic Syndrome 3 Misclassified as Type 1 Diabetes Mellitus

**DOI:** 10.1210/jcemcr/luae120

**Published:** 2024-08-06

**Authors:** Suhaib Radi, Lujain Bashamakh, Hayfa Mandourah, Sarah Alsharif

**Affiliations:** College of Medicine, King Saud Bin Abdulaziz University for Health Sciences, Jeddah 22384, Saudi Arabia; Department of Medicine, King Abdullah International Medical Research Center, Jeddah 22384, Saudi Arabia; Department of Internal Medicine, Division of Endocrinology, King Abdulaziz Medical City, Ministry of the National Guard-Health Affairs, Jeddah 22384, Saudi Arabia; Department of Internal Medicine, King Faisal Specialists Hospital and Research Center, Jeddah 22384, Saudi Arabia; Department of Internal Medicine, King Faisal Specialists Hospital and Research Center, Jeddah 22384, Saudi Arabia; Department of Family Medicine, Ministry of National Guard-Health Affairs, King Abdulaziz Medical City, Jeddah 22384, Saudi Arabia

**Keywords:** type 2 diabetes mellitus (T2DM), type 1 diabetes mellitus (T1DM), abdominal obesity–metabolic syndrome (AOMS), *DYRK1B* gene

## Abstract

Age is no longer the most important differentiating feature between type 1 and type 2 diabetes, as obesity and metabolic syndrome are on the rise in the pediatric population. Here we present a case of a 30-year-old male individual initially diagnosed with uncontrolled type 1 diabetes mellitus (T1DM) since the age of 15, and treatment with high insulin doses has been unsuccessful. He was later identified as having abdominal obesity–metabolic syndrome 3 (AOMS3) based on strong family history and the presence of insulin resistance features. AOMS3 is characterized by early-onset coronary artery disease, central obesity, hypertension, and diabetes. Early detection of this condition is crucial to implement timely interventions and preventing the onset of complications.

## Introduction

Diabetes mellitus is categorized mainly into 2 primary types: type 2, which accounts for 90% to 95% of cases, and type 1, constituting approximately 5% of the cases (1). Despite certain similarities between the 2 types, their pathophysiology and management significantly differ. Age used to be the major distinguishing feature between the 2 types; however, this is no longer the case given the rising prevalence of obesity among adolescents and young adults, which poses considerable challenges in classifying diabetes in this cohort and plays a major role in treatment decisions and subsequently determining the course of family counseling and screening. Moreover, developing type 2 diabetes (T2DM) at a young age has been associated with a worse prognosis and a higher risk of complications (2). Here we present a rare genetic cause for T2DM in a young patient misdiagnosed as having type 1 diabetes mellitus (T1DM) for many years.

## Case Presentation

**Figure 1.**
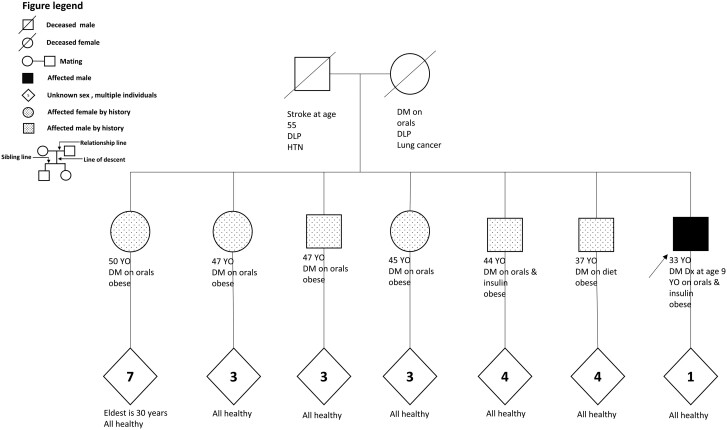
Square/circle with a diagonal line: deceased male/fema. Dotted square/circle: Affected individual with metabolic syndrome but no genetic testing available. Blackened square/circle: Affected individual with metabolic syndrome and positive genetic testing. Diamond: Unknown sex, multiple individuals.

A 30-year-old Saudi man has been diagnosed with diabetes mellitus since the age of 15, based on hyperglycemic symptoms and elevated hemoglobin A1c (HbA1c). He was labeled as having T1DM because of his young age upon diagnosis. Despite being on multiple daily injections (MDI) of insulin with a total daily dose exceeding 200 units, his diabetes remained uncontrolled, leading to complications that included nephropathy, proliferative diabetic retinopathy, peripheral neuropathy, and erectile dysfunction. He was referred to our center a year ago for further evaluation of his uncontrolled diabetes. His history includes hypertension, he is a smoker with a sedentary lifestyle, and he never had diabetic ketoacidosis. Upon a thorough review of the patient's family history, it was discovered that all 6 siblings have been affected with diabetes and obesity (Fig. 1). Four of the siblings are on oral medications, one is on insulin, and one is just on a diet. The mother had T2DM, dyslipidemia, and died of lung cancer. The father had stroke, dyslipidemia, and hypertension. The mother had 5 siblings, all of whom were in good health. In contrast, of the father’s 8 siblings, 4 had suffered strokes previously. The third generation, which comprises 25 children, the eldest age 30 years, are all healthy so far.

## Diagnostic Assessment

On physical examination at the first visit to our center, his blood pressure was 137/95 and his weight was 97 kg with a body mass index (BMI) of 34.4 kg/m^2^ (normal [ref]: 18.5-24.9). Acanthosis nigricans was observed on his neck and underarms, while the rest of the physical examination showed no abnormalities. Laboratory results at diagnosis were as follows: HbA1c 13% (ref: < 6.0 [119 mmol/mol (ref: < 42)]), random blood glucose 30.4 mmol/L (ref: < 11 [547.2 mg/dL (ref: <200)]), C-peptide of 123 pmol/L (ref: 252-1176 [0.37 ng/mL (ref: 0.76-3.6)]). Upon his visit to our center a year ago, his laboratory evaluation showed a HbA1c of 9.4% [79 mmol/mol], random blood glucose reading of 15.8 mmol/L [284.7 mg/dL], albumin to creatinine ratio of 7.4 mg/mmol (ref: 0-2.5 [65.49 mg/g (ref: < 30)]), total cholesterol of 5.56 mmol/L (ref: < 5.17 [215 mg/dL (ref: < 200)]), triglyceride of 2.96 mmol/L (ref: < 1.7 [262 mg/dL (ref: < 150)]), low-density lipoprotein cholesterol (LDLC) of 2.97 mmol/L (ref: < 3.36 [114.8 mg/dL (ref: < 130)]), and high-density lipoprotein cholesterol (HDLC) of 1.13 mmol/L (ref: > 1.5 [43.7 mg/dL (ref: ≥ 40)]). The thyroid function test, serum creatinine, and electrolytes were normal.

Given that many features in this patient did not fit with his label of T1DM; including his insulin resistance and strong family history, further investigations to classify his diabetes were done. A repeat C-peptide was normal at 538 pmol/L (1.624 ng/mL), the anti-glutamic acid decarboxylase (anti-GAD) antibody came back negative, and genetic testing was sent for him.

## Treatment

Over the next 3 months, his insulin was tapered down and eventually stopped and he was started gradually on metformin, pioglitazone, gliclazide, dapagliflozin, and semaglutide (Table 1).

**Table 1.** Chronological order of laboratory results and interventions

Timing	HbA1c	Random blood glucose	Interventions
At the age of diagnosis, 15 years old	13%/119 mmol/mol (ref: <6%/<42 mmol/mol)	30.4 mmol/L/547.2 mg/dL (ref: <11 mmol/L/<200 mg/dL)	Started on multiple daily insulin injections with titrated doses until he reached a total daily dose of 225 units
At age of 30 (first presentation to our clinic, time 0)	9.4%/79 mmol/mol (ref: <6%/<42 mmol/mol)	15.8 mmol/L/284.7 mg/dL (ref: <11 mmol/L/<200 mg/dL)	After confirming normal C-peptide and negative Antibodies, his total daily dose was deceased to 150 units and metformin, semaglutide were added
6-week follow-up	NA	10.4 mmol/L/187.2 mg/dL (ref: <11 mmol/L/<200 mg/dL)	Insulin aspart was stopped, insulin glargine was decreased to 50 units daily, dapagliflozin was added
3-month follow-up	8.6%/70 mmol/mol (ref: <6%/<42 mmol/mol)	7.9 mmol/L/142.2 mg/dL (ref: <11 mmol/L/<200 mg/dL)	Insulin glargine was stopped, gliclazide and pioglitazone were added
6-month follow-up	7%/53 mmol/mol (ref: <6%/<42 mmol/mol)	5.6 mmol/L/100.8 mg/dL (ref: <11 mmol/L/<200 mg/dL)	Patient kept on same regimen (metformin, pioglitazone, gliclazide, dapagliflozin, and semaglutide)

Abbreviations: HbA1c, glycosylated hemoglobin; ref, reference value.

## Outcome and Follow-Up

The patient demonstrated significant improvement within the next few months. His self-monitored blood glucose (SMBG) readings at home showed fasting levels below 7.7 mmol/L (138.8 mg/dL), and postprandial levels below 11.1 mmol/L (200 mg/dL). His repeated biochemical results showed a fasting blood glucose of 6.6 mmol/L (118.9 mg/dL), HbA1c of 7% (53 mmol/mol), LDLC of 3.7 mmol/L (143.1 mg/dL), and an improved albumin to creatinine ratio of 2.1 mg/mmol (18.58 mg/g). Genetic testing came back positive for *DYRK1B* mutation, indicating abdominal obesity–metabolic syndrome type 3 (Table 2).

**Table 2.** Genetic testing results

Gene (Isoform)	Phenotype MIM number (mode of inheritance)	Variant	Zygosity
DYRK1B (NM_004714.3)	615812 (AD)	C.137G>A P.(ARG46HIS) chr19:40321350	Heterozygous

The coding exons of more than 20 000 genes were enriched using Roche/KAPA sequence capture technology and sequenced on an illumine system (next-generation sequencing, NGS); 9 out of 21 bioinformatic in silico programs predict a pathogenic effect for this variant.

## Discussion

Contrary to common belief, T2DM is not solely confined to older adults. The growing rates of obesity locally and globally and the more sedentary lifestyle has increased the incidence of T2DM in younger population. The high prevalence rates of both T2DM (16.4%) and obesity (28.7%) in Saudi Arabia emphasize the critical need to effectively address these health issues (3, 4). One study described a case of a 21-year-old patient initially thought to have T1DM who required high dose of insulin, which raised the suspicion for insulin resistance, especially with presence of acanthosis nigricans. Subsequent testing revealed negative anti-GAD antibody and elevated C-peptide level. Eventually, the patient was discovered to have Alström syndrome associated with insulin resistance and T2DM, which responded positively to insulin sensitizers, resulting in cessation of insulin (5). This case underscores the importance of reconsidering diabetes diagnoses based on clinical features and biomarkers, irrespective of age, to avoid potential misclassifications and to optimize patient care.

There are some clues that should trigger searching for alternative diagnoses rather than T1DM in a young patient. These include family history, increased weight, clinical evidence of insulin resistance with high doses, sedentary lifestyle, acanthosis nigricans, and presence of metabolic syndrome. The Framingham offspring study discovered that the likelihood of developing T2DM was 3.5 times higher among offspring with one parent affected by diabetes, and the likelihood rises to 6 times higher for those with both parents affected (6). Moreover, obesity by itself, particularly abdominal fat, plays a significant role in the onset of T2DM, leading to its occurrence at an earlier age (7). In the last 5 years, obesity prevalence rates in several developing countries were more than 15% in children and adolescents aged 5 to 19 years; 41.8% in Mexico, 22.1% in Brazil, 22.0% in India, and 19.3% in Argentina (8).

In a study conducted on a sample of 3036 Iranian adolescents aged 10 to 19 years, consisting of 1413 boys and 1623 girls, it was found that the prevalence of metabolic syndrome was significantly higher among overweight (defined based on the standardized percentile curves of BMI suggested for Iranian children and adolescents as ≥ 95th percentile of BMI for age and sex) participants compared to those at risk for overweight (defined as ≥ 85th to < 95th percentile of BMI for age and sex) and those with normal weight (defined as < 85th percentile of BMI for age and sex). Among boys, 41.1% of overweight participants had metabolic syndrome, while the rates for those at risk for overweight and those with normal weight were 11.4% and 3.0%, respectively (*P* < .01). Rates were also similar for female participants (9). In a separate study involving participants aged 15 to 39 years from 204 countries and territories during the period of 1990 to 2019, it was found that high BMI was identified as the predominant risk factor contributing to early-onset T2DM (10).

Based on the National Cholesterol Education Program ATP III criteria, patient had metabolic syndrome and genetic testing could confirm the diagnosis (11). T2DM develops when insulin resistance is coupled with non-autoimmune β-cell failure. Hyperinsulinism, typically triggered by insulin resistance, contributes to the upregulation of fatty acid production. This, in turn, leads to heightened triglyceride levels, reduced HDLC levels, and compromises glucose transporters (12, 13). Identifying this at an early stage and implementing targeted therapy for insulin resistance could enhance patient outcomes. We believe the young age of our patient upon diagnosis was the major contributor to misclassifying his condition as T1DM. Delving into the family history and thoroughly examining the patient's pedigree could offer valuable insights for a more precise diagnosis. The fact that all the patient's siblings have obesity and have been diagnosed with T2DM, coupled with his mother being known to have T2DM, emphasizes the significance of incorporating family history into the diagnostic process. However, initial low C-peptide level could be another contributing factor. Nonetheless, it is essential to keep in mind that C-peptide levels may not be accurately interpretable during acute hyperglycemia. Severe hyperglycemia can lead to glucotoxicity and pancreatic shock and prevent insulin secretion, resulting in underestimation of C-peptide level. Thus, it is recommended to assess C-peptide level after correcting the hyperglycemia (14).

A group of features that includes abdominal obesity, low levels of HDL, high triglycerides, high blood pressure, and elevated fasting blood glucose is called abdominal obesity–metabolic syndrome (AOMS) (1). It is a contributory risk factor for both cardiovascular disease (CVD) and diabetes mellitus (15-17). The etiology is complex, determined by the interplay of both genetic and environmental factors including diet and physical inactivity (18). AOMS2 has been mapped to chromosome 17p12 and AOMS4 is caused by mutation in the CELA2A gene on chromosome 1p36 (19). AOMS3, an autosomal dominant condition, is caused by a mutation in the *DYRK1B* gene on chromosome 19q13 (19). This mutation recognized to be associated with a syndrome of central obesity, hypertension, T2DM, and early-onset coronary artery disease (20). The elevated risk of CVD appears to be linked to various risk factors and insulin resistance associated with metabolic syndrome, rather than being solely attributed to obesity. A particular study underscored that individuals characterized by obesity without metabolic syndrome did not exhibit a significantly elevated susceptibility to CVD or diabetes (15).

Conversely, those individuals presenting with obesity and metabolic syndrome had a 10-fold and 2-fold escalation in the risk of diabetes mellitus and CVD, respectively (15). The approach to manage metabolic syndrome involves 2 main strategies to target the underlying causes, the first is reducing weight through lifestyle modifications, and the second is to address cardiovascular risk factors if they persist despite lifestyle changes (21).

A number of variant mutations to *DYRK1B* gene found to co-segregate with abdominal obesity syndrome have been reported in the literature (22, 23). Other variants of *DYKR1B* mutation were found and were associated with other unrelated conditions, including myoepithelial tumor and inborn genetic disorder (23). To the best of our knowledge, the variant we report here has not been described in the literature of AOMS so far (24). The allele frequency of this variant in the general population has not been documented (25). This is the first time we detect it in our internal database in a heterozygous state. Considering the available information, the variant is classified as a variant of uncertain significance.

Identifying AOMS3 as the cause of diabetes in our patient was very crucial and allowed us to switch his 15-year-long insulin treatment, which was unsuccessful, to other oral and injectable agents with proven cardiovascular and weight benefits, resulting in significant improvement in his glycemic and metabolic markers and better treatment satisfaction.

In conclusion, this case highlights the importance of considering diabetes types other than type 1 in young adults. It emphasizes the need for comprehensive history-taking and examinations, guided by appropriate investigations, to enhance the likelihood of achieving an accurate and timely diagnosis, thereby improving patient management. Insulin resistance should be suspected, especially in cases requiring high insulin doses or exhibiting features of insulin resistance such as acanthosis nigricans. Successful management of AOMS and T2DM necessitates proper education and adherence to medication, healthy diet, and physical activity.

## Learning points

- Abdominal obesity–metabolic syndrome is a genetic cause for insulin resistance and diabetes presenting at young age and can be misdiagnosed as type 1 diabetes.
- Clues to diagnosis of abdominal obesity–metabolic syndrome include presence of acanthosis nigricans, strong family history, and high insulin requirements (> 1 unit/kg/day).
- Insulin sensitizers, such as metformin and thiazolidinediones, are an important part of management of insulin resistance and can help in lowering insulin requirements.
- Weight loss using healthy lifestyle +/− glucagon like peptide-1 (GLP-1) receptor agonists can significantly improve metabolic syndrome and hyperglycemia.

## Data Availability

Original data generated and analyzed for this case report are included in this published article.

## Abbreviations

anti-GAD: anti-glutamic acid decarboxylase
AOMS: abdominal obesity–metabolic syndrome
BMI: body mass index
CVD: cardiovascular disease
HbA1c: glycosylated hemoglobin
HDLC: high-density lipoprotein cholesterol
LDLC: low-density lipoprotein cholesterol
ref: reference value
T1DM: type 1 diabetes mellitus
T2DM: type 2 diabetes mellitus
